# Related Factors for Unfavorable Disease Course in Patients with Crohn’s Disease: An Observational Retrospective Study

**DOI:** 10.3390/diagnostics13020273

**Published:** 2023-01-11

**Authors:** Dong Yoon Han, Myung-Won You, Chi Hyuk Oh, Seong Jin Park

**Affiliations:** 1 Department of Radiology, Kyung Hee University College of Medicine, Kyung Hee University Hospital, Seoul 02447, Republic of Korea; 2 Department of Internal Medicine, Kyung Hee University College of Medicine, Kyung Hee University Hospital, Seoul 02447, Republic of Korea

**Keywords:** Crohn’s disease, inflammatory bowel disease, disease progression, follow-up studies, prognosis

## Abstract

Background: Crohn’s disease (CD) manifests a heterogeneous clinical spectrum and disease course, and it is challenging to predict the disease outcome based on initial presentation. Objective: To analyze the long-term disease course and factors leading to poor prognosis of CD. Methods: In total, 112 patients with CD who were initially diagnosed and treated at our institution from January 2009 to August 2020 were included. We analyzed their clinical data, disease characteristics according to the Montreal classification, and the endoscopic and computed tomography (CT) examinations at the initial visit and at 2-year, 5-year, and last follow ups. We categorized the disease course into the following four categories: remission, stable, chronic refractory, and chronic relapsing. Significant factors associated with a poorer prognosis were analyzed. Results: The median follow-up period was 107 (range, 61–139) months. Complicated disease behavior increased slightly over the follow-up period (20.5% to 26.2%). An unfavorable disease course was defined as chronic refractory (19.6%) and relapsing (16.1%) courses. The 2-year disease characteristics were significant factors for unfavorable disease course, and the combination of 2-year perianal disease and 2-year moderate-to-severe CT activity could predict unfavorable disease course with the highest accuracy (0.722; area under the curve: 0.768; *p* < 0.0001). Conclusions: One-third of the patients with CD showed an unfavorable disease course (35.7%), and 2-year disease characteristics were significant factors for an unfavorable disease course.

## 1. Introduction

Crohn’s disease (CD) is a chronic inflammatory condition of the gut of unknown etiology that presents with a clinical course of relapse and remission and commonly affects young people. Approximately 20–30% of CD patients might undergo a nonprogressive or indolent course, while the majority of patients with CD might present with progressive and destructive bowel inflammation and develop cumulative structural damage to the bowel over time [1,2]. However, the clinical spectrum and disease course of CD are frequently heterogeneous and often difficult to predict based on the initial presentation.

Due to the heterogeneous disease pattern of CD, the stratification of patients according to several morphologic phenotypes has been proposed for the determination and optimization of treatment planning, assessment of disease outcome, and communication between physicians and patients. The current Montreal classification [3] is widely used in both research and clinical practice; however, it is rather a cross-sectional assessment and has a major drawback in that it shows instability in assessing disease extent, location, and behavior over a period of time [4]. Hence, a stratification of clinical subgroup according to a longitudinal disease course is necessary to reflect changing disease patterns over time. Knowledge about the long-term changes in the disease course and factors influencing prognosis can be used as a basis for treatment planning, optimal follow up, and assessment of prognosis. 

Therefore, we aimed to investigate the disease course and related factors for unfavorable disease course during long-term treatment and follow up in patients with CD. The primary goal was to evaluate the course of disease in patients with CD followed up for over 7 years at our institution. Additionally, we attempted to identify prognostic factors related to the disease course.

## 2. Materials and Methods

### 2.1. Patients

**Figure 1:**
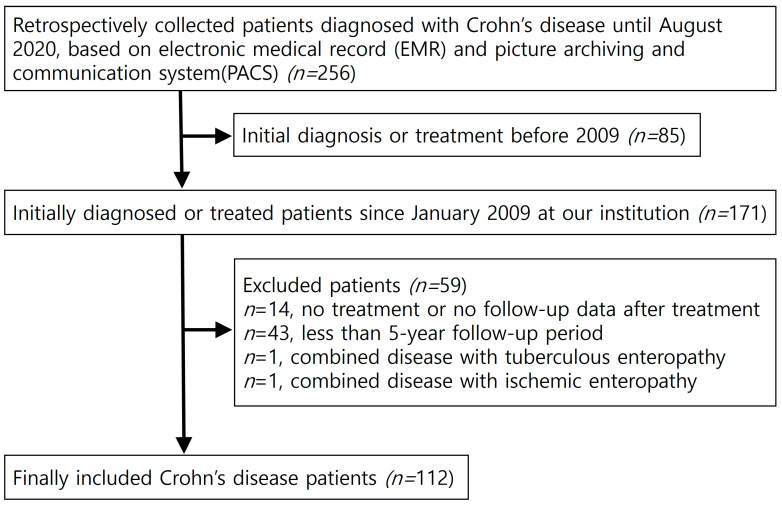
Flow chart of patient selection.

This study was approved by the Institutional Review Board (IRB) of our institution (IRB file No: 2022-12-042), and the requirement for informed consent was waived. We searched the electronic medical records (EMRs) and picture archiving and communication system (PACS) images of patients diagnosed with CD from January 2000 to August 2020. The diagnosis of CD was determined according to clinical evaluation and a combination of biochemical, radiological, endoscopic, and histological investigations [5,6]. Patients who were initially diagnosed with CD or treated regularly at our institution were consecutively selected as candidates for our study. Of the 256 patients identified, 85 patients were excluded, as their initial records were from before 2009, and the available data were incomplete. Hence, 171 patients who were initially diagnosed with or treated for CD since January 2009 at our institution remained. Among these patients, 59 patients were excluded for the following reasons: (1) no identifiable follow-up data after treatment (*n* = 14), (2) follow-up period <5 years (*n* = 43), (3) difficult-to-evaluate disease pattern or activity due to combined disease with tuberculous enteropathy (*n* = 1), and (4) combined disease with ischemic enteropathy (*n* = 1). Lastly, a total of 112 patients with CD were included in this study (Figure 1).

### 2.2. Clinical Data Analyses

We investigated the patients’ clinical information, including age, sex, presence of bowel symptoms, Crohn’s disease activity index (CDAI), history of prior bowel or perianal operation, nonsmoker or former/current smoker, presence of family history of inflammatory bowel disease, disease duration from the initial to the last visit, and presence of extra-intestinal manifestations (e.g., arthritis, Raynaud phenomenon, sclerosing cholangitis, or ankylosing spondylitis). Information regarding treatment, such as use of systemic steroids at initial diagnosis; use of immunosuppressants (IMS), including azathioprine, mercaptopurine, and methotrexate; usage and type of biologics (including infliximab, adalimumab, golimumab, vedolizumab, ustekinumab, tofacitinib, and filgotinib); time point of initial use of biologics since diagnosis (no use, initial use, first use within 2-year, between 2-year and 5-year, or after 5-year follow up); and single or combination therapy of IMS with biologics, were recorded. A “regimen change” was defined as a change of IMS to biologics, “change of biologics” was defined as change of one type of biologics to another type of biologics, and “dose intensification” was defined as a dose increase of 2-fold that of the usual dose. 

### 2.3. Radiologic and Endoscopic Analyses of Disease Pattern

Two abdominal radiologists (with 13 and 8 years of experience) reviewed the contrast-enhanced computed tomography (CECT) images and recorded the CT activity scores. Although several CT scoring systems have been adapted in other studies [7,8,9], none have been credibly validated, and the grading of disease severity was only partially quantified without cutoff values of grades. Therefore, we generated a modified CT scoring system similar to the previously reported MR activity scoring system [10], with reference to CT features evaluated on other reported CT scoring systems [11]. The following CT features were assessed for each patient: wall thickness; wall enhancement with enhancement pattern; total length of the diseased segment; presence of comb sign (vasa recta prominence); and the presence of any complications, including abscess, fistula, or severe stenosis. Stricturing and penetrating diseases were defined according to consensus recommendations [12]. According to the sum of the total score measured with these features, one of the four categories of no activity or mild, moderate, or severe activity was determined (Table 1). In cases involving more than one lesion, the lesion with the highest activity score was selected for categorization. The scoring of CT activity was independently performed by each reviewer, consensus scoring of CT activity was performed with a 2-week interval, and the interreader agreement was assessed. The disease pattern was evaluated according to the Montreal classification [3] and Paris classification for patients with age ≤18 [13], based on CECT and endoscopic features. Disease location was classified into one of three categories: L1, ileal; L2, colic; and L3, ileocolic disease. Disease behavior was classified into one of three categories: B1, non-stricturing non-penetrating inflammatory; B2, stricturing; B3, penetrating, and combined stricturing and penetrating disease. For pediatrics (age ≤ 18), combined structuring and penetrating disease was classified as B2B3. Upper gastrointestinal tract involvement (L4 lesion) and presence of perianal lesions (P) were separately recorded. L4a and L4b lesions were separately recorded for pediatrics. In the case of no visible active or chronic lesion, disease location and behavior were recorded as “none.” A gastroenterologist evaluated endoscopic activity using simple endoscopic score of Crohn’s disease (SES-CD) system retrospectively. He was blinded to the associated clinical and radiologic data. 

### 2.4. Classification of Disease course during the Entire Follow-Up Period

The disease course was classified into the following four categories: remission, stable, chronic refractory, and chronic relapsing. “Remission” was defined as more than two of three categories among clinical remission, endoscopic remission, and CT activity score 0–1 at the last follow up. “Clinical remission” was defined as a decrease in the severity of bowel symptoms or CDAI < 150. “Endoscopic remission” was defined as the achievement of complete mucosal normalization [14]. An individual case was classified as “remission” if the disease activity decreased at the last follow up without any residual activity after modification of medical treatment, such as a regimen change or dose optimization, even if there was an increase in the severity of disease activity during the follow up. “Stable” was defined as an individual in clinical remission with residual endoscopic activity or residual CT activity grade normal to mild. “Chronic refractory” was defined as wax and wane, but persistent clinical symptoms and radiologic/endoscopic activities without remission. “Chronic relapsing” was defined as more than one relapse in bowel symptoms or radiologic/endoscopic activities for each treatment regimen. “Relapse” was defined as an aggravation of bowel symptoms, CT, or endoscopic activities leading to the requirement of more intensive medical and/or surgical treatment after achieving remission. We modified these definitions from a previous report [15], adding radiological and endoscopic activities to overcome mismatched cases between clinical symptoms and radiological/endoscopic findings, especially in cases with small bowel lesions. 

### 2.5. CT Techniques

CT examinations were performed using one of the following multidetector CT scanners: 16-channel Lightspeed from GE healthcare (*n* = 111), 64-channel Brilliance (*n* = 66), 128-channel Ingenuity (*n* = 81) from Philips Healthcare, and 64-channel Aquilion from Toshiba (*n* = 62) at four time points (initial visit and 2-year, 5-year, and last follow ups). The scanning parameters were as follows: a peak voltage of 120 kVp; a tube current–time product of 150–200 mAs with automated tube current modulation; a 2.5~5 mm slice thickness with a 3 mm reconstruction interval; a field of view of 300–380 mm; a gantry rotation time of 0.5–0.6 s; detector configuration of 0.625 mm; z-axis coverage of 24, 40, and 40 mm; pitch 0.9, 0.7, and 0.8 s; table speeds of 43.2, 47.5, and 63.8 mm per second; and a single breath-hold helical acquisition time of 9–10 s for 16-, 64-, and 128-channel CT exams. Among the total CT exams (*n* = 335), two-thirds were small bowel enterographies (*n* = 241, 72%), and the remaining one-third was conventional abdomen–pelvis CT (*n* = 92, 27%). The CT protocol for small bowel enterography was as follows: patients were asked to drink 1500 mL of 3% polyethylene glycol solution (Harprep, Pharmbio, Korea) 45 min before scanning for maximum distension of the small bowel. For contrast enhancement, 120 mL of nonionic contrast medium (Iomeprol 350, Bracco Imaging) was injected intravenously at a rate of 2 mL/s, followed by flushing with 20 mL of saline. The scanning delay was 60 s for the portal phase, and additional coronal reformatted images were obtained.

### 2.6. Statistical Analyses

Comparisons of differences in categorical and continuous data were performed using Fisher’s exact probability test or Wilcoxon rank sum test. The association of variables with disease course was investigated using univariate and multivariate logistic regression analyses. All variables with *p*-values < 0.1 from univariate regression analysis were included in the multivariate model. Adjustment with the Firth’s method was used for multivariate regression analysis. Receiver operating characteristic (ROC) curve analyses were performed using the selected disease-related variables in the multivariate logistic regression. To perform time-to-event analyses, we used the log-rank test or Cox proportional hazards model. Statistical analyses were performed using SPSS version 26 (IBM, New York, NY, USA), SAS version 14.3 (SAS Institute Inc., Cary, NC, USA), and MedCalc version 14.8.1 (Ostend, Belgium). *p* < 0.05 was considered as a significant result.

## 3. Results

### 3.1. Analyses of Disease Course during the Long-Term Follow-Up Period

#### 3.1.1. Disease Course and Outcomes

The median follow-up period was 107 months (range, 61–139 months). Among the four categories of disease course, the rates were as follows: 27.7% (31/112) for remission, 36.6% (41/112) for stable, 19.6% (22/112) for refractory, and 16.1% (18/112) for relapsing. Therefore, the well-controlled disease course constituted 64.3% (72/112), and the unfavorable disease course constituted 35.7% (40/112). In all, 16 patients underwent bowel surgery (14.3%), and 9 patients underwent perianal surgery (8%). The median time to relapse was 103.5 months (range, 41–139 months), and the median time to bowel surgery was 104 months (range, 1–139 months).

#### 3.1.2. Changes in Disease Patterns

**Figure 2:**
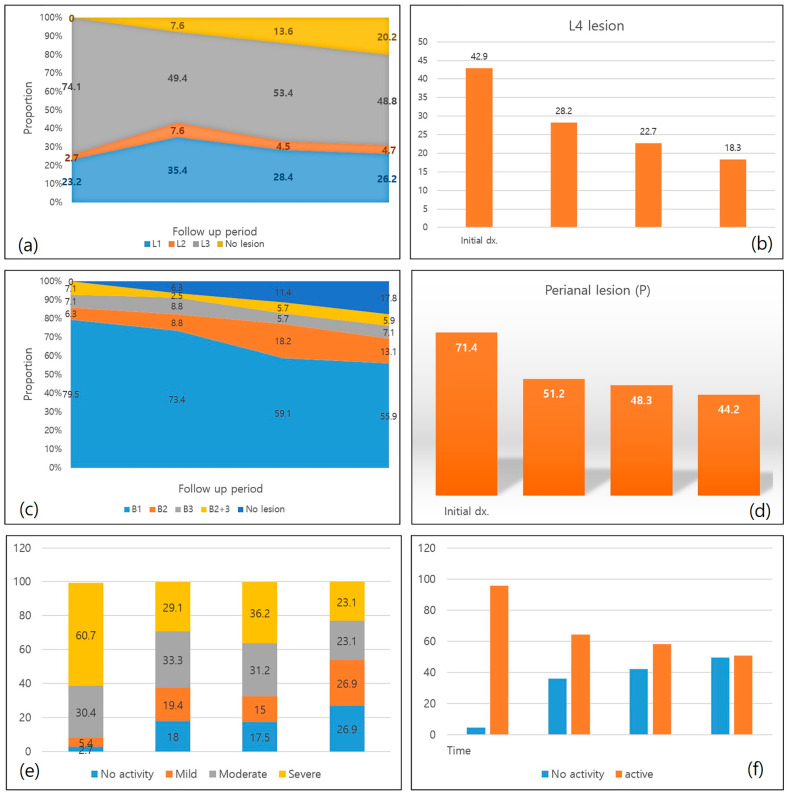
Changes in disease pattern. (**a**) Disease location from initial diagnosis to the last follow up; (**b**) percentage of presence of L4 lesion (upper gastrointestinal tract involvement); (**c**) disease behavior from initial to the last follow up; (**d**) percentage of perianal disease; (**e**) disease activity evaluated on CT images; (**f**) disease activity evaluated on endoscopy.

Figure 2 shows the changes in disease patterns over time. The disease location was relatively stable, but the proportion of L3 disease decreased, while the proportion of no lesions increased. Complicated disease behavior (B2-4) slightly increased from 20.5% to 26.1%, but 17.8% showed no lesions at the last follow up. Both L4 lesions and perianal disease gradually decreased, as did active endoscopic lesions. CT activity scores also gradually decreased from 91.1% to 46.2% at the last follow up, and the kappa value of interreader agreement for CT activity scoring was 0.51 (standard error: 0.07), indicating moderate agreement (Figure 2).

### 3.2. Analyses of Disease Characteristics for Unfavorable Disease Course

Comparison of clinical and disease characteristics between well-controlled and unfavorable disease course

The mean age of the study population was 24.73 ± 9.4 (range, 12–51) years, and 82.1% (92/112) of patients were male. Systemic steroids were used in only 5.4% (6/112) of patients at the initial visit. The regimen for the induction of remission was either steroid with or without sulfasalazine (80.4%) or biologics (infliximab or adalimumab, 19.6%). Most of the patients received immunosuppressants (IS) (96.4%, 108/112), more than one type of biologic (79.5%, 89/112), and combination therapy (64.3%, 74/112). The time points of the first use of biologics were different in each group; patients with a well-controlled course mostly started biologics later than the initial visit, while those with an unfavorable course mostly started from the initial visit (34.2%, *p* = 0.012). A higher percentage of patients experienced dose intensification (40% vs. 6.94%) and switched from one type of biologic to another (65% vs. 12.5%) in the unfavorable course group compared to those in the well-controlled disease course group (Table 2).

At the initial visit, the most frequent disease location was the ileocolon (L3, 74.1%), the B1 type of disease behavior was most represented (79.5%), and the CT activity score was mostly moderate (30.4%) or severe (60.7%). L4 lesions were present in 42.9% (48/112), and perianal disease was present in 71.4% (80/112). There were no significant differences in these variables between the two groups at the initial visit. At the 2-year follow up, the overall distributions of disease location and behavior were similar to those at the initial visit. However, the percentage of complicated disease behavior (B2-3) (32.26% vs. 12.5%, *p* = 0.045), perianal lesions (68.75% vs. 39.58%, *p* = 0.013), and moderate or severe grades of CT activity (82.76% vs. 48.84%, *p* < 0.001) were higher in the unfavorable disease course group than those in the well-controlled disease course group at 2-year follow up (Table 3). Regarding endoscopic activity, initial SES-CD scores mostly indicated moderate activity (56/111, 50%) and the distribution of SES-CD scores showed no difference between the two groups (*p* = 0.950). SES-CD scores at 2-year follow up were mostly indicative of remission (SES-CD score <4, 56/111, 50%), and there was no difference in the distribution of SES-CD scores between the two groups (*p* = 0.923).

Thirty-one patients aged ≤18 years were evaluated according to the Paris classification system. The L4a lesion was present in 29% (9/31) and the L4b lesion in 25.8% (8/31) at baseline, with no significant difference in percentage of those between the two groups (*p* = 0.897). The proportion of L4 lesions decreased at 2-year follow up, showing L4a lesions in 15% (3/20) and L4b lesions in 15% (3/20).

### 3.3. Significant Factors for Unfavorable Disease Course

In the multivariate logistic regression analysis, 2-year complicated disease behavior (OR, 3.87; 95% CI, 1.14–13.16), 2-year perianal disease (OR, 3.43; 95% CI, 1.24–9.51), and 2-year moderate-to-severe grades of CT activity (OR, 4.95; 95% CI, 1.55–15.83) were the significant factors among the disease characteristics for unfavorable disease course (Table 4). Moreover, change in biologic (OR, 12.38; 95% CI, 4.54–33.75), dose intensification (OR, 7.81; 95% CI, 2.59–23.51), and combination therapy (OR, 3.31; 95% CI, 1.30–8.43) were the significant factors among the treatment-related variables for unfavorable disease course. All variables related to the initial disease pattern showed no significant odds ratios for disease course.

### 3.4. ROC Analyses for Unfavorable Disease Course Using Selected Disease Parameters

**Figure 3:**
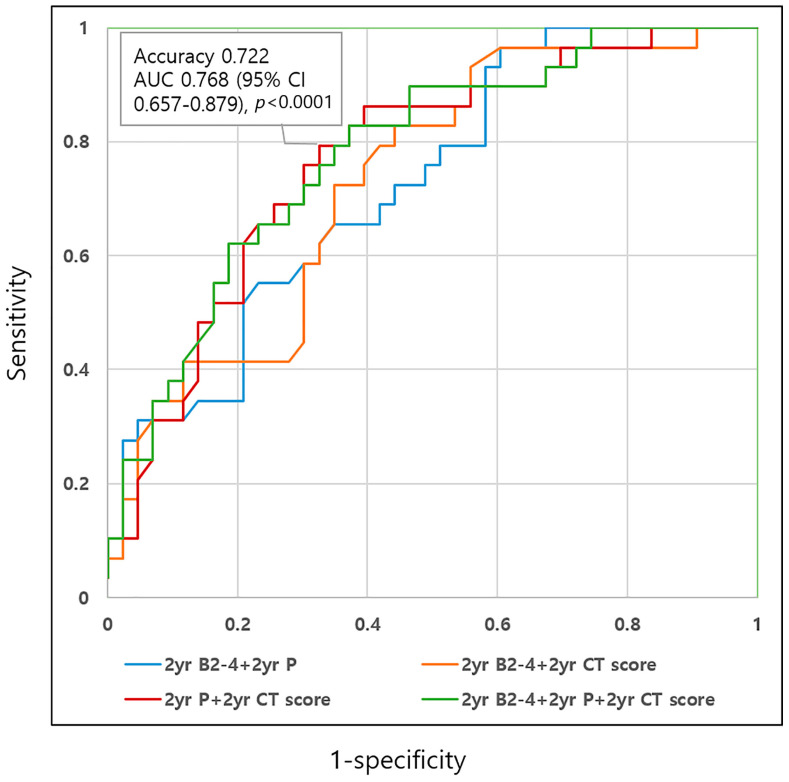
ROC analysis for unfavorable disease course using selected disease parameters. Combining 2-year perianal disease with 2-year moderate-to-severe CT activity scores showed the highest accuracy (0.722) and AUC (red line, 0.768, *p* < 0.0001), which is comparable to those of the combination of all the three variables (2-year B2-3, 2-year perianal disease, and 2-year moderate-to-severe CT scores, green line, 0.779, *p* < 0.0001).

**Figure 4:**
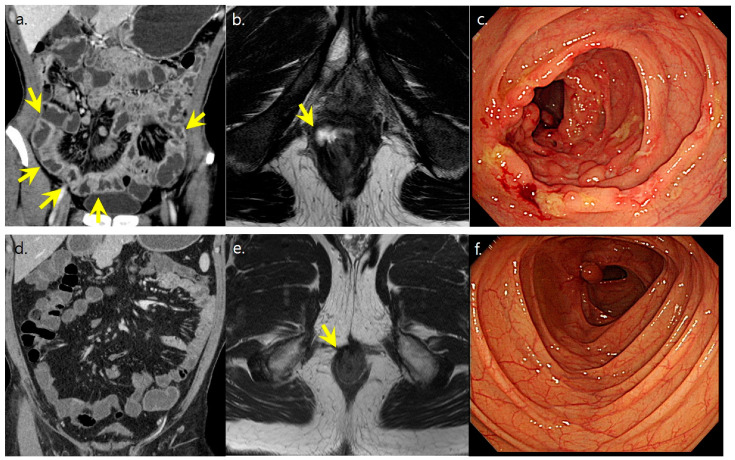
A 36-year-old male with Crohn’s disease showing well-controlled disease course. (**a**,**b**) Initial CT and MR showed active inflammation in the entire ileum and colon with perianal abscess. (arrows) CT scores was graded as severe activity. (**c**) Initial SES-CD score was graded as severe with multiple ulcers, exudates, erythema, and cobble stone appearance in the colons. (**d**,**e**) Two-year follow-up CT showed resolved active inflammation in both bowel loops and perianal region (arrows in (**e**)), and CT scores was graded as no activity. (**f**) Patient was in clinical and endoscopic remission at the last follow up.

**Figure 5:**
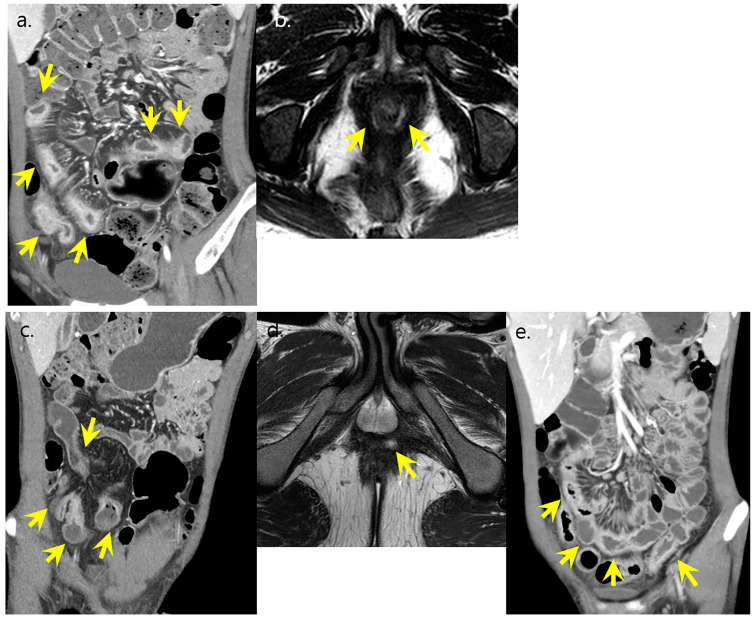
A 22-year-old male with Crohn’s disease showing unfavorable disease course. (**a**,**b**) Initial CT and MR showed severe active inflammation in entire ileum and ileocolon with active perianal lesion. (arrows) CT scores were graded as severe activity. (**c**,**d**) Follow-up exams after 2 years showed persisting active bowel inflammation and perianal lesion. (arrows) (**e**) CT activity was graded as severe due to persistent active bowel inflammation at the last follow up (arrows).

We analyzed the ROC curves for unfavorable disease course using the selected disease-related factors (Table 5, Figure 3). The three disease-related factors for unfavorable disease course were as follows: 2-year B2-4, 2-year perianal disease, and 2-year moderate-to-severe CT scores, all of which were used for the generation of diagnostic prediction models. Among the models, 2-year perianal lesion with 2-year moderate-to-severe CT scores model showed the highest diagnostic accuracy (0.722) and area under the curve (AUC, 0.768; *p* < 0.0001), which was comparable to those of the combination of the three variables (accuracy: 0.708; AUC: 0.779; *p* < 0.0001) (Figure 4 and Figure 5).

## 4. Discussion

In this study, patients with CD showed gradually decreasing L4 lesions, perianal disease, active endoscopic lesions, and moderate-to-severe CT activity; however, approximately half of the patients showed persistent moderate-to-severe CT (46.2%) and endoscopic activities (50.6%). The disease locations were relatively stable over time, with the majority consisting of L1 and L3 lesions. The proportion of complicated disease behavior slightly increased over time (initial: 20.5% to last: 26.2%), but was insignificant compared to previous reports of 42.7~50.7% [16,17] and a more recent study reporting 31% [18]. Although the initial proportion of complicated disease behavior was slightly higher than that reported in previous studies (20.5% vs. 17%–18.6%) [16,18], it was lower for the last follow up than reported in these studies (26.2% vs. 31%–42.7%). Both Ye et al.’s study [17] and our study show improved clinical course in Korean patients with CD than in Western patients. 

In this study, approximately two-thirds of patients showed a well-controlled disease course, while one-third showed an unfavorable course, such as chronic refractory or relapsing disease. Moreover, the 2-year disease patterns, rather than the initial manifestation, were significant factors for unfavorable disease course. These findings correlate with those of previous studies. Beaugerie et al. also reported that the clinical prognosis can be better determined during the course of the first 2 years than with the clinical parameters present at the time of onset [19]. Another study on an inception cohort from Denmark showed that relapse rates within 2 years after the diagnosis significantly correlated with those in the following 5 years, whereas no useful clinical factors were identified within a year of diagnosis for the prediction of clinical progress [20]. Apart from these studies from Western countries, a study conducted with a Japanese cohort reported that the prediction of an 11- to 15-year clinical course is possible using clinical factors during the year following treatment and also by determining the effectiveness of the initial treatment [21]. Taken together, it might be stated that the treatment response or effectiveness after initial treatment determines the long-term disease course better than the initial clinical or radiologic presentations at the time of disease onset. We additionally analyzed diagnostic prediction models using the 2-year disease-related factors. As a result, we found that 2-year perianal disease with moderate-to-severe CT activity scores predicted unfavorable disease course with the highest accuracy. Quaz et al. also reported that perianal disease is associated with disease severity. [22] Perianal disease is a well-known factor associated with poor prognosis [18]. However, Song et al. emphasized that perianal CD (pCD) was not a risk factor for intestinal resection, behavior progression, or CD-related hospitalization, and therefore, it possesses a less significant clinical impact on the overall outcome of pCD in Asian patients [23]. This result correlates with ours. We found that the persistent perianal disease at 2-year follow up was one of the significant factors associated with an unfavorable disease course, whereas the baseline perianal disease itself was not. Additionally, disease severity determined with CT activity scores is a significant factor for disease course in the combination of perianal disease. Disease activity assessment was usually performed using a CD activity index, based on patients’ symptom reporting or phenotypic classifications such as multisegmental B2 or B3 diseases in previous studies [24,25]. In this study, we used both radiologic and endoscopic disease activity scorings to achieve objective and quantitative assessments of disease activity. Although MRI grading is a more validated method for the assessment of disease activity, CT and MRI shows comparable highly accurate grading estimates according to meta-analysis [11]. At our institution, most of the included patients with CD underwent CT exams during disease monitoring, which allowed us to generate a CT-based disease activity scoring system to assess radiologic disease activity in a quantitative manner. This CT-based disease activity score can be used for disease monitoring and the prediction of disease course based on our results.

A total of 31 patients were pediatrics (age ≤ 18) in our study population, and their regimen for the induction of remission was either steroid with sulfasalazine/antibiotics or biologics (infliximab/adalimumab). According to new ECCO-ESPHGAN guidelines, exclusive enteric nutrition (EEN) is recommended as a first line for the induction of remission in mild-to-moderate luminal CD [26]. However, it is not an available option at our institution; therefore, no pediatric CD patient was treated with EEN. This might be because gastroenterologists manage most of the CD patients, including both adults and pediatrics, who visit the specialized IBD center managed by the gastroenterology department at our institution. 

As for the strength of our study, we assessed the long-term data relating to disease monitoring and treatment in CD patients. We summarized the longitudinal information and categorized it into several types to define the factors that are useful for clinical decision-making. 

This study has a few limitations. First, this is a retrospective study and poses inherent flaws. Moreover, several patients had missing or incomplete clinical information, such as smoking status, family history, CD activity index, and extraintestinal manifestation. Second, a relatively small number of patients were analyzed. Due to incomplete old clinical data, we confined the eligible patients to those who were diagnosed after 2009. Third, the follow-up periods varied among the included patients, and not all patients at the initial visit underwent CT or endoscopic exam at the next follow up. Therefore, fewer data were obtained during the 2-year, 5-year, and last follow ups compared to those of the initial visits. Fourth, heterogeneous CT techniques were used to obtain CT scans of the included patients during the long-term follow-up period. Fifth, the CT scoring in this study was not fully validated. It is important to quantitively analyze CT exams, but there is no credibly validated CT scoring system; therefore, we developed a modified CT scoring system with reference to previous response assessment studies [11,27]. Moreover, endoscopic activity was assessed using SES-CD scores, which are more validated, to supplement the CT activity scoring system. Sixth, included patients received heterogeneous treatments. Most of the patients received IS (96.4%), which included azathioprine, 6-mercaptopurine, and methotrexate, although most received azathioprine (93.8%). Moreover, approximately 80% of the included patients received biologics, which included seven types.

## 5. Conclusions

Patients with CD with initially active disease showed gradual improvement in disease activities during the long-term follow up, with only 26.2% of complicated disease behavior at the end of the follow-up period. However, one-third of patients showed an unfavorable disease course, such as chronic refractory or relapsing diseases, and the 2-year follow-up disease characteristics, but not the initial manifestations, were significant factors for unfavorable disease course.

## Figures and Tables

**Table 1:** Modified CT scoring system.

	None: 0	Mild: 1	Moderate: 2	Severe: 3
Wall thickness	≤3 mm	4–5 mm	6–7 mm	≥8 mm
Wall enhancement (compared to adjacent vessels)	Normal	Mild: minor increase, but less than vessels	Moderate: moderate increase, but less than vessels	Severe: marked increase, similar to vessels
Enhancement pattern	Normal	Homogenous	Mucosal ^₤^	Layered ^€^
Disease length	None	5 cm	5–15 cm	>15 cm
Comb sign	No	Yes		
None: no signs of active disease. Mild: total scores ≤6, no features with score 3. Moderate: total scores 7–10 or presence of any one feature ≥3. Severe: total scores ≥11 or presence of at least one complication including abscess, fistula, or stenosis.				

Homogenous enhancement pattern is defined as a one-layered bowel wall with enhancement. ^₤^ Mucosal enhancement pattern is defined as a two-layered bowel wall with inner layer enhancement. ^€^ Layered enhancement pattern is defined as a three-layered bowel wall with inner and outer layer enhancements and hypo-enhancement of the middle layer.

**Table 2:** Comparison of clinical characteristics of CD patients between well-controlled and unfavorable disease course.

Variables	Overall	Well-Controlled Disease Course (*n* = 72) (Remission+ Stable)	Unfavorable Disease Course (*n* = 40) (Refractory+ Relapse)	*p*-Value (<0.05)
Age at diagnosis, mean (±sd)	24.73 (±9.4)	25.15 ± 9.84	23.98 ± 8.63	0.709
Male (%)	92 (82.1)	60 (83.33)	32 (80)	0.797
Smoking (%)				0.382
Nonsmoker	26 (23.2)	17 (70.83)	9 (60)	
Former	2 (1.8)	2 (8.33)	0	
Current	11 (9.8)	5 (20.83)	6 (40)	
Unknown	73 (65.2)			
Family history (%)				1.000
No	23 (20.5)	15 (93.75)	8 (100)	
Yes	1 (0.9)	1 (6.25)	0	
Unknown	88 (78.6)			
Prior operation history (%)	6 (5.4)	3 (4.2)	3 (7.5)	0.664
Prior perianal operation history (%)	24 (21.4)	14 (19.44)	10 (25)	0.631
Systemic steroid at initial visit (%)	6 (5.4)	3 (4.92)	3 (8.11)	0.669
Induction of remission (%)				0.14
Steroid (per oral) ± sulfasalazine/antibiotics	90 (80.4)	61 (84.7)	29 (72.5)	
Biologics (IFX or ADA)	22 (19.6)	11 (15.2)	11 (27.5)	
Immunosuppressants (IS) (%)	108 (96.4)	69 (95.8)	39 (97.5)	1.000
Time points of first usage of Biologics (%)				**0.012**
Initial	26 (29.2)	13 (25.4)	13 (34.2)	
Within 2-year f/u	24 (26.9)	14 (27.4)	10 (26.3)	
Within 5-year f/u	23 (25.8)	16 (31.4)	7 (18.4)	
After 5-year f/u	16 (17.9)	8 (15.7)	8 (21)	
Combination therapy (%)	72 (64.3)	39 (55.71)	33 (82.5)	**0.006**
Switch of IS to biologics (%)	62 (55.4)	37 (51.39)	25 (62.5)	0.322
Switch of one to another biologic (%)	35 (31.3)	9 (12.5)	26 (65)	**<0.0001**
Dose intensification (%)	21 (18.8)	5 (6.94)	16 (40)	**<0.0001**

IFX; infliximab, ADA; adalimumab. Bold numbers indicate *p* < 0.05.

**Table 3:** Comparison of disease characteristics of CD patients between well-controlled and unfavorable disease course.

Variables	Overall	Well-Controlled Disease Course (*n* = 72) (Remission+ Stable)	Unfavorable Disease Course (*n* = 40) (Refractory+ Relapse)	*p*-Value (<0.05)
Initial visit				
Location at initial visit(*n*, %)				0.839
L1	26 (23.2)	18 (25)	8 (20)	
L2	3 (2.7)	2 (2.78)	1 (2.5)	
L3	83 (74.1)	52 (72.22)	31 (77.5)	
L4 lesion at initial visit (*n*, %)	48 (42.9)	28 (38.89)	20 (50)	0.319
^α^ L4a	9/31 (29)	6/18 (33.3)	3/13 (23)	
^β^ L4b	8/31 (25.8)	4/18 (22.2)	4/13 (30.7)	0.897
Behavior at initial visit (*n*, %)				1.000
B1	89 (79.5)	57 (79.17)	32 (80)	
B2	7 (6.3)	5 (6.94)	2 (5)	
B3	16 (14.2)	10 (13.8)	6 (15)	
^γ^ B2B3	1/31 (3.2)	0/18	1/13 (3.2)	
Perianal disease at initial visit (*n*, %)	80 (71.4)	49 (68.06)	31 (77.5)	0.383
CT activity score at initial visit (*n*, %)				0.137
None	3 (2.7)	2 (2.82)	1 (2.5)	
Mild	6 (5.4)	6 (8.45)	0 (0)	
Moderate	34 (30.4)	24 (33.8)	10 (25)	
Severe	68 (60.7)	39 (54.93)	29 (72.5)	
SES-CD score at initial visit (mean± SD)	9.97 ± 5.57	9.88 ± 5.52	10.15 ± 5.72	0.804
SES-CD score at initial visit (*n*, %)				0.95
Remission (<4)	9 (8)	6 (8)	3 (7.5)	
Mild (4–6)	24 (21.4)	15 (21.1)	9 (22.5)	
Moderate (7–15)	56 (50)	37 (52.1)	19 (47.5)	
Severe (>15)	22 (19.6)	13 (18.3)	9 (22.5)	
Second-year follow up				
Location at 2nd-year f/u				0.345
No lesion (*n*, %)	6 (7.9)	5 (10.42)	1 (3.23)	
L1	28 (35.4)	19 (39.58)	9 (29.03)	
L2	6 (7.6)	4 (8.33)	2 (6.45)	
L3	39 (49.4)	20 (41.67)	19 (61.29)	
L4 lesion at 2nd-year f/u (*n*, %)	22 (28.2)	10 (21.28)	12 (38.71)	0.124
^α^ L4a	3/20 (15)	2/12 (16.6)	1/8 (12.5)	
^β^ L4b	3/20 (15)	1/12 (8.3)	2/8 (25)	0.785
Behavior at 2nd-year f/u (*n*, %)				**0.045**
No lesion	5 (6.3)			
B1	58 (73.4)	B1: 42 (87.5)	B1: 21 (67.74)	
B2	7 (8.9)	B2-3: 6 (12.5)	B2-3: 10 (32.26)	
B3	9 (11.4)			
Perianal disease at 2nd-year f/u (*n*, %)	41 (51.3)	19 (39.58)	22 [68.75]	**0.013**
CT activity score at 2nd-year f/u (*n*, %)				**<0.001**
None	13 (18.1)	10 (23.26)	3 (10.34)	
Mild	14 (19.4)	12 (27.91)	2 (6.9)	
Moderate	24 (33.3)	16 (37.21)	8 (27.59)	
Severe	21 (29.2)	5 (11.63)	16 (55.17)	
SES-CD score at 2nd-year f/u (mean± SD)	4.36 ± 4.86	4.67± 5.27	3.82 ± 4.06	0.422
SES-CD score at 2nd-year f/u (*n*, %)				0.923
Remission (<4)	56 (50)	34 (56.6)	22 (64.7)	
Mild (4–6)	18 (16.1)	12 (20)	6 (17.6)	
Moderate (7–15)	14 (12.5)	10 (16.6)	4 (11.7)	
Severe (>15)	6 (5.4)	4 (6.7)	2 (5.8)	

SES-CD, simple endoscopic score for Crohn’s disease; SD, standard deviation. ^α^ Upper gastrointestinal tract involvement, proximal to the Treitz ligament according to the Paris classification. ^β^ Upper gastrointestinal tract involvement, distal to the Treitz ligament according to the Paris classification. ^γ^ Both penetrating and structuring disease according to the Paris classification. Bold numbers indicate *p* < 0.05.

**Table 4:** Multivariate regression analyses for unfavorable disease course.

	Exp(B)	95% Confidence Interval (CI)	*p*-Value
Initial L4 lesion	1.6	0.71–3.60	0.255
Initial disease behavior: B2-3	0.99	0.35–2.80	0.990
Initial perianal lesion	1.49	0.58–3.84	0.412
Initial moderate-to-severe CT activity score	3.65	0.56–23.83	0.176
Initial SES-CD score	1.01	0.94–1.08	0.82
2-year L4 lesion	2.33	0.83–6.56	0.109
2-year disease behavior: B2-3	**3.87**	**1.14–13.16**	**0.030**
2-year perianal disease	**3.43**	**1.24–9.51**	**0.017**
2-year moderate-to-severe CT activity score	**4.95**	**1.55–15.83**	**0.007**
2-year SES-CD score	0.96	0.87–1.05	0.41
Use of immunosuppressants (IS)	1.18	0.13–10.58	0.885
First biologics use within 2 years	0.48	0.21–1.07	0.072
Biologics change	**12.38**	**4.54–33.75**	**<0.0001**
Dose intensification	**7.81**	**2.59–23.51**	**<0.0001**
Combination therapy	**3.31**	**1.30–8.43**	**0.012**

Adjusted by age, sex, prior operation, and prior perianal operation. Bold numbers indicate *p* < 0.05.

**Table 5:** ROC curve for sensitivity analyses for unfavorable disease course.

	Sensitivity	Specificity	Accuracy	AUC ^€^ (CI)	*p*-Value
2yr disease behavior: B2-3	0.621 (18/29)	0.628 (27/43)	0.625 (45/72)	0.625	0.07
2yr perianal disease	0.793 (23/29)	0.558 (24/43)	0.653 (47/72)	0.673	0.007
2yr moderate-to-severe CT activity score	0.759 (22/29)	0.628 (27/43)	0.681 (49/72)	0.686	0.004
2yr B2-4+ 2yr P	0.655 (19/29)	0.651 (28/43)	0.653 (47/72)	0.724	0.0002
2yr B2-3+ 2yr CT score	0.724 (21/29)	0.651 (28/43)	0.681 (49/72)	0.731	0.0001
**2yr P+ 2yr CT score**	**0.793** **(23/29)**	**0.674** **(29/43)**	**0.722** **(52/72)**	**0.768**	**<0.0001**
2yr B2-3 + 2yr P + 2yr CT score	0.759 (22/29)	0.674 (29/43)	0.708 (51/72)	0.779	<0.0001

2yr, 2 year; B2-3, complicated disease behavior; P, perianal disease; AUC, area under the curve; CI, confidence interval. ^€^ Adjusted by sex, age, prior operation history, prior perianal operation history, and initial variables. Bold numbers indicate the model with highest accuracy.

## Data Availability

The data presented in this study are available on request from the corresponding author. The data are not publicly available due to privacy.
